# Canadian Occupational Performance Measure: Benefits and Limitations Highlighted Using the Delphi Method and Principal Component Analysis

**DOI:** 10.1155/2022/9963030

**Published:** 2022-03-02

**Authors:** Jean-Michel Caire, Sabrina Maurel-Techene, Thierry Letellier, Margit Heiske, Sarah Warren, Arnaud Schabaille, Florent Destruhaut

**Affiliations:** ^1^Occupational Therapy Training Institute in Toulouse, URU Evolsan, University of Toulouse, France; ^2^Occupational Therapy Training Institute in Toulouse, ToNIC, UMR Inserm, University of Toulouse, France; ^3^URU Evolsan, Paul Sabatier University, Toulouse, France; ^4^URU Evolsan Laboratory, University of Toulouse, France; ^5^Occupational Therapy Training Institute, Toulouse, France; ^6^Occupational Therapy Training Institute, Saint Sébastien de Morsent, France; ^7^Paul Sabatier University, CHU Toulouse, URU Evolsan, France

## Abstract

**Introduction:**

The objective of this study was to establish a baseline of current use in practice of the Canadian Occupational Performance Measure (COPM) by consulting 33 expert French occupational therapists, who trained in this method between 2012 and 2017 and use of the COPM with their clients. The areas of health intervention are pediatrics, psychiatry, neurology, and geriatrics. An email invitation to participate in the research was therefore sent to 113 occupational therapists. We received 33 responses.

**Methods:**

A novel mixed method study combined a Delphi method with a lexical analysis of experts' responses and principal component analysis (PCA).

**Results:**

In the last Delphi round, the consensus of the expert group was attained on 31 benefits and 1 limitation, confirming the generally positive influence of the COPM in French health services. *Discussion.* The COPM was clearly identified as a tool that supports occupational therapy clinical reasoning, facilitates team decision-making for care pathways, and enables people with disabilities and health conditions to make decisions for their care.

**Conclusion:**

The Delphi study revealed that the COPM appears to be well adapted to French culture and should be widely incorporated into preregistration training.

## 1. Introduction

Human beings need to engage in activities to maintain and develop their abilities, their life in society, and their health. Occupational therapists' principal goal is to support people to engage in meaningful occupations, especially when they have disabilities or health conditions [1]. The Canadian Occupational Performance Measure (COPM) is an outcome measure that evaluates changes in daily occupations and satisfaction for a person over time. The COPM administration consists of a semistructured interview that enables the client and therapist to evaluate and to prioritize everyday issues that restrict their participation in everyday living [2]. According to their own perceptions, the client rates their occupations in terms of the level of importance and their satisfaction [2]. This tool facilitates the setting of rehabilitation goals and the development of an intervention plan. Its use is consistent with client-centered practice and is useful in rehabilitation research [3]. Because the outcome measure is founded on the principles of the Canadian Model of Occupational Performance and Engagement (CMOP-E) that is focused on the occupational perspective, rather than being based on a pathology, it can be used with a wide range of clients. The aim of the therapeutic relationship in this context is to facilitate personal and social change through the lens of meaningful occupation. Decision-making power is therefore rightfully located with the client in order that they can express their priorities and expectations in relation to their treatment [4]. However, the health model is still primarily disease-centered, and French occupational therapists are more oriented towards a “bottom up” than “top down” intervention. To change direction, the 2010 Reforms in preregistration occupational therapy training in France clearly emphasize the learning of conceptual occupational models, which includes the CMOP-E and therefore a possible alternative to the use of the COPM.

In France, the use of the CMOP-E has been widely integrated into occupational therapy practice via Continual Professional Development (CPD) training. However, to date, no studies on the use of this tool by occupational therapists have been carried out. The objective of this research is to participate in the change in practice in France towards an approach client-centered and occupation-centered. The operational objective is to establish a baseline of the use of the COPM with a group of expert occupational therapists who were trained between 2012 and 2017 and are considered an expert in this study, with any occupational therapist who has completed an additional 28 hours of training of COPM, use of the COPM with their clients, and the concept of habilitation. This study enabled us to identify the benefits and limitations of the use of the practice of COPM, based on the feedback of a group of expert occupational therapists in France. The results of this study may be a response to the health department's request to participate in individualized health coaching [5] and to engage in an international dynamic of the definition of occupational therapy included in the occupation sciences with a common international language.

## 2. Materials and Methods

### 2.1. Study Approach

The Delphi technique [6] is a research method that aims to achieve a consensus via the judgement of experts. The consultation of experts facilitates the development and prioritization of areas that can lead to actions to resolve social issues, and it is widely used across many specialist professions working in social contexts [6–7]. This technique is comprised of 4 essential stages: (1) constitution of an expert group, (2) development of a questionnaire, (3) consultation process, and (4) tabulation and analysis of results. It is based on two key principles: anonymity of results and independence of analysis [7]. The objective is to identify convergence of expert opinion and achieve a consensus on the subject being studied. This is a very useful technique to bring together the opinions of experts around a subject, to support novel thinking or transformations, in domains including health and rehabilitation [8]. The process can take two to four rounds, depending on the number of responses and level of consensus. Consensus is considered as sufficient once agreement between experts reaches 70-80% [6].

### 2.2. Participants and Recruitment

The selection criteria for this study were as follows: state qualified occupational therapists in France, completion of CPD training of 28 hours training in the use of CMOP-E between 2012 and 2017, regularly use of the COPM with their clients (people with disabilities and health conditions, but also their caregivers), French-speaking, and no connection to political group or commercial companies to avoid external influence and pressure. It was not required to be used systematically COPM for each client to take the context in France and the predominantly disease-centered approach. The participants were recruited by email from a list of occupational therapists having completed CPD at the French national occupational therapy association. The first consent to participate in the research was sent through the ANFE association in a confidential manner. An email invitation to participate in the research was therefore sent to 113 occupational therapists, who had completed COPM training. The areas of health intervention are pediatrics, psychiatry, neurology, and geriatrics. In all, there were 33 positive responses (29.2% response rate) that were received from a wide range of participants across France.

### 2.3. Data Collection and Analysis

**Figure 1:**
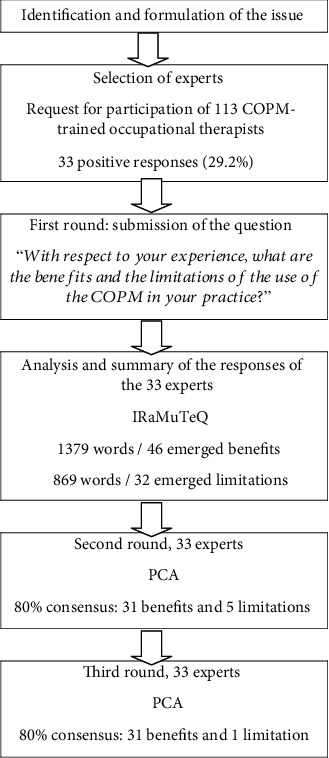
Summary of the Delphi technique combined with IRaMuTeQ and PCA.

**Figure 2:**
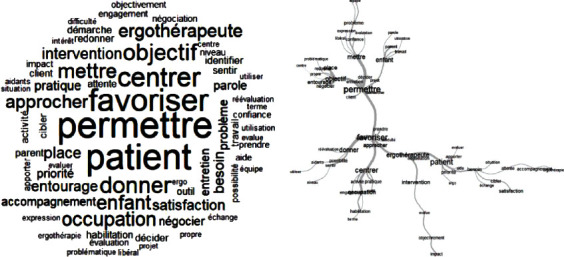
IRaMuTeQ analyses of COPM benefits. (a) Word cloud. (b) Similarity network.

**Figure 3:**
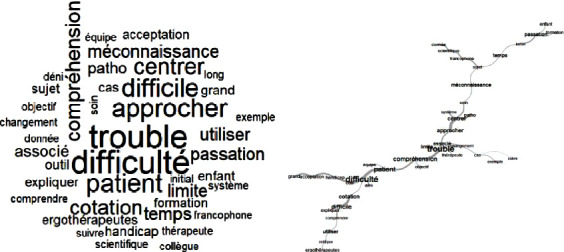
IRaMuTeQ analyses of COPM limitations. (a) Word cloud. (b) Similarity network.

**Figure 4:**
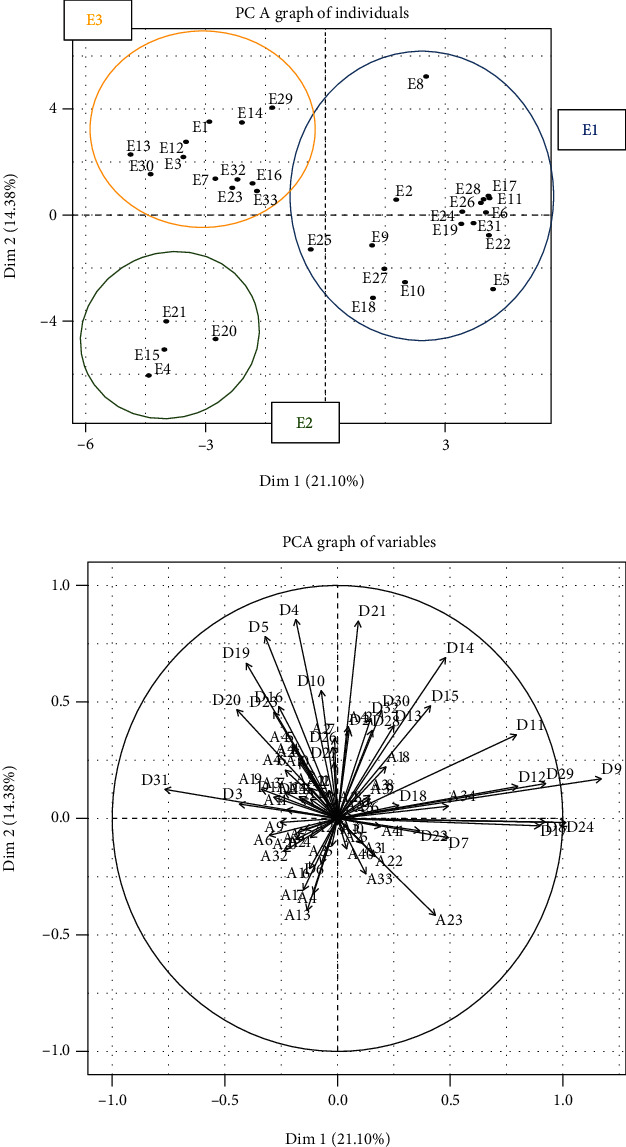
Principal component analysis (PCA) of the second consultation. (a) Projection of experts. (b) Projection of benefits (a) and limitations (d).

**Figure 5:**
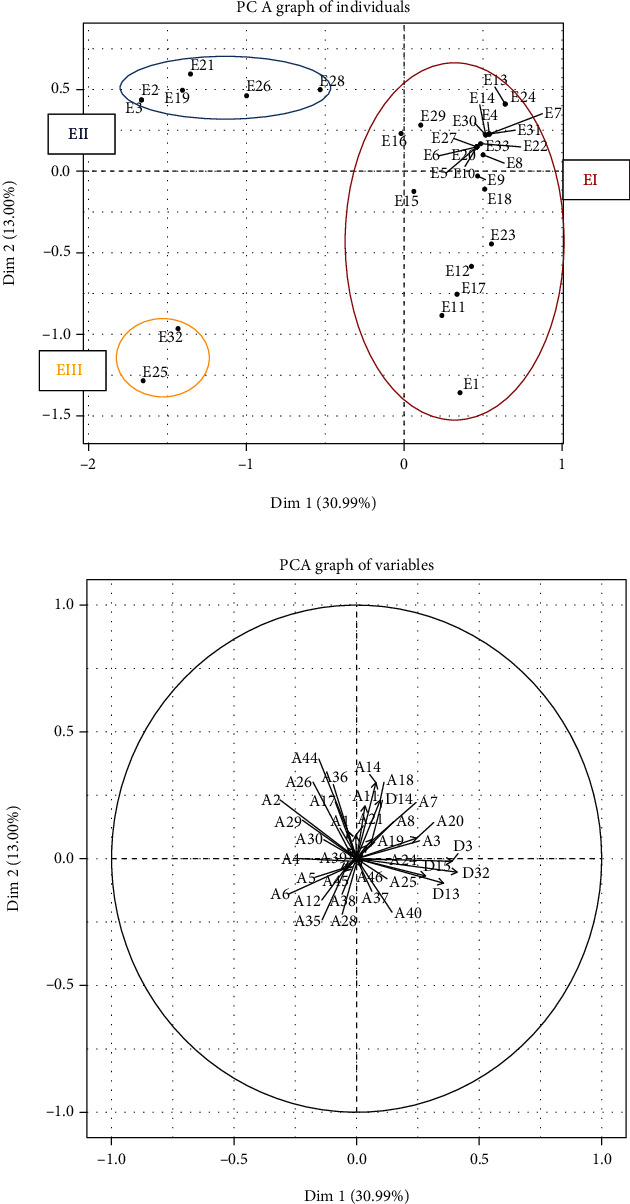
Principal component analysis (PCA) of the third consultation. (a) Projection of experts. (b) Projection of benefits (a) and limitations (d).

The Delphi technique begins with an open question, which is the essential starting point for the process of exploring the problem [9]. This study began by asking the participants a double question: “With respect to your experience, what are the benefits and the limitations of the use of the COPM in your practice?” This is the only question that was asked to the occupational therapists. This question was sent by email to each occupational therapist, who had three weeks to reply. Although there is no strict limit to the length of a reply, suggested 10 to 15 words per response were proposed, in order to facilitate synthesis of ideas. To maintain anonymity, each reply was numbered and collected together without identifying data (Figure 1). The body of replies was then analyzed using the textual analysis software IRaMuTeQ. The similarity analysis of a textual matrix facilitates the description of lexical classes, profiles of specific aspects, and groupings [10]. This data analysis, based on graph theory, points out cooccurrences between words, generates lexical commonalities, and highlights similar and redundant words (Figures 2 and 3). The second consultation of experts was carried out using elements from the first round. In order not to influence the decisions of experts, the items were randomly generated into a list. For each list element, the experts were asked to select a level of importance, using a four-point Likert scale from 1 “do not agree at all” to 4 “completely agree.” This kind of scale is widely used in the context of occupational and social psychology and in education science [11]. Following the Delphi technique, only items with at least 80% of agreement between experts were retained for the third round. Here, the experts were asked to rate these items once again, this time with either “agree” or “do not agree.” The final list was then generated with the items having a consensus of 80% or more. For rounds two and three, principal component analysis (PCA) was used to capture the profiles of the participants' opinions. This analysis permitted the reduction of a multidimensional graph (corresponding to the number of items) to a two-dimensional graph, representing the expert profiles. By decreasing the number of variables and rendering the information less redundant, maximum variability between the expert's profiles was conserved. The PCA graphs (Figures 4(a) and 5(a)) reveal clusters of points, which thus permitted the definition of groups of experts having similar lines of thinking. Moreover, the projection of the different items (Figures 4(b) and 5(b)) revealed the general themes in the thinking of these occupational therapists concerning their use of COPM and its general use in today's health system in France. In fact, in these graphs, the length and the direction of the vector represent the discriminative importance of the respective item, and they influence the position of the experts' profiles on the PCA graph. Indeed, the greater the length of the vector, the further towards its direction the expert is positioned on the PCA graph.

## 3. Results

### 3.1. Characteristics of the Participants

**Figure 6:**
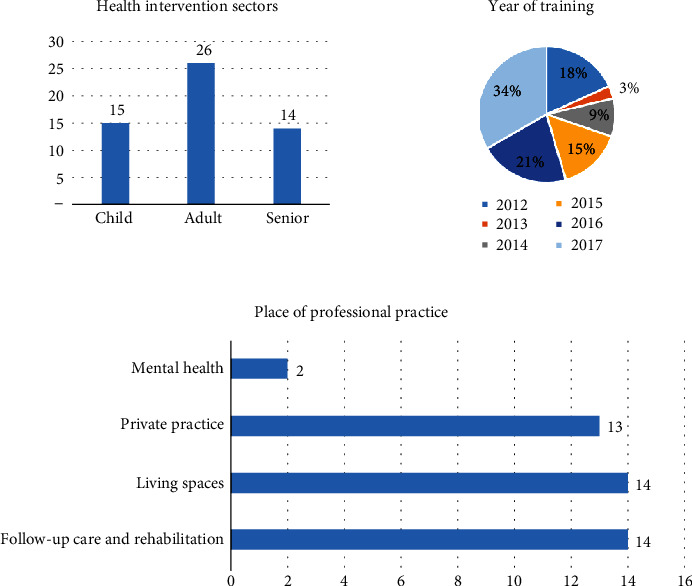
Demographic elements of the expert population.

A total of 33 experts participated, of which 79% (*n* = 26) were women and 21% (*n* = 7) were men. In all, 72.7% (*n* = 24) of the occupational therapists had completed state registered occupational therapy training before 2010, and 27.3% (*n* = 9) completed it after the program reforms of 2010. In relation to domains of practice, 42.4% (*n* = 14) practiced in postdischarge and rehabilitation, 42.4% (*n* = 14) in the community, 39.4% (*n* = 13) in independent practice, and 6% (*n* = 2) in mental health. 45.4% (*n* = 15) practiced in pediatrics, 78.7% (*n* = 26) with adults, and 42.4% (*n* = 14) with seniors. The year of CPD training in CMOP-E and the use of the COPM were thus as follows: 2012 (*n* = 6, 18%), 2013 (*n* = 1, 3%), 2014 (*n* = 3, 9%), 2015 (*n* = 5, 15%), 2016 (*n* = 7, 21%), and 2017 (*n* = 11, 33%). The occupational therapists were from different regions in France. The average caseload numbers per year for this group of 33 occupational therapists were 68. All 33 occupational therapists participated in the three rounds of consultation (Figure 6).

### 3.2. First Consultation of Experts

The word cloud (IRaMuTeQ) of benefits was created using the first body of data (1379 words, 33 questionnaires for the first round) (Figure 2). It was conceived based on the frequency of words and highlighted the importance of the following words: to enable, patient, to foster, to centre, to give, to approach, objective, occupational therapist, to put intervention, occupation, priority, place, and caregivers. It demonstrates the positive influence of the use of the COPM in practice. The COPM influences importantly the development of the occupational therapy intervention by taking into account the patient and his or her caregivers. Prioritization of the patient's objectives featured strongly in the narratives of the expert occupational therapists. The COPM directs the occupational therapist towards the question of daily occupations of patients and their satisfaction with them. The analysis of the benefits of the COPM reveals a commonality in key words such as enables and fosters (Figure 2(a)). These two words emphasize more the idea of “care” (help, take care of) than that of “cure” (carry out, treat). The textual references to “foster” evidence a therapeutic context, the method, and form of which link directly back to the use in practice of the COPM. The textual branch: foster, approach, center, practice, occupation, and habilitation links back to the epistemological and conceptual approach of the occupational therapist in their use of the COPM. The textual branch: “foster,” “occupational therapist,” “negotiation,” “patient,” “need,” “expectation,” “treatment,” and “satisfaction” reveals the therapeutic framework of the interview, which emphasizes the expression of needs, expectations, and patient satisfaction to formulate the intervention of the occupational therapist. The textual branch: “occupational therapist,” “negotiation,” “intervention,” “evaluation,” “objectively,” and “impact” describes the role that the experts give to the occupational therapist as an assessor of the daily impact of disability. The textual branch: “foster,” “give,” “possibility,” and “re-evaluation” brings into play the use in interventions of practical elements arising from assessment using the COPM. The use of the COPM ends with “decide” and “put” in place “team” therapeutic plans (Figure 2(b)). The link is made between the expression of difficulties and the “team”, underlining that the COPM can serve as an interprofessional tool.

The limitation word cloud relies on the same principles of frequency of words in the text. For the first round, the initial body of data for limitations is 869 words from 33 questionnaires (Figure 3(a)). The cluster shows the importance of the words: impairment, difficulty, lack of knowledge, understanding, patient, use of, implementation, rating, time, and center. For the experts, these limitations seem to arise mainly from difficulties in understanding on the part of the patients, but also from a lack of knowledge of the COPM and an approach centered on pathologies. Implementation and rating are also identified as factors of limitations. The diagram of commonalities (Figure 3(b)) clearly demonstrates how the difficulties are centered around 2 distinct axes of four words: impairment, understanding, patient, and difficulty. The first axis deals with the patient's “difficulties” which impact on their “rating” as “difficult” to “understand” and “explain” and “utilise” with “colleagues.” The occupational therapist is at the limits of these correlations, being directly impacted by this group of limitations. In connection with the “patient” and “understanding”, the multidisciplinary team may also impede the use of the COPM. “Acceptance and “denial” of the “patient” are also limiting factors for the use of the COPM, according to the experts. The other axis is that of the organization and the setting of the interventions. The concept of impairment can steer us towards “pathology” and, together with “systems” of “treatment” which are also conceived around “pathology”, this can limit the facilitation of “change” centered around daily activities of living and occupation and the beneficial elements of the COPM. The lack of compelling French language scientific data about it increases this feeling of ignorance. The experts' words have been captured and detailed in order to be able to classify them and ensure the synthesis of items by similarity, organizing them into semantic categories, frequencies of words, and distribution patterns [12]. After analysis of these first round, 46 items were isolated in relation to benefits and 32 items for limitations (Table 1).

### 3.3. Second Consultation of experts

All 33 experts participated in the second round of consultation. The consensus was 80% agreement. The data are presented in order of importance according to the frequency of responses obtained. Occupational therapists identified 32 interests (Table 2(a)) and only 5 important limitations (Table 2(b)) for using CORM in their practice.

Principal component analysis (PCA) revealed that although the occupational therapists participating in the Delphi study were largely in agreement over the items (51/78), three groups of experts could be differentiated from each other (Figure 4(a)). Following their choices for some of the items (11/46 benefits and 16/32 limitations), three lines of thinking were highlighted (Figure 2(a)), as follows:

Group E1 (17/33): limitations brought this group of occupational therapists together. In this group, they were more or less in accord about the limitations of the COPM, with regard to the organizational systems of French health institutions (D2) and the lack of knowledge of rehabilitation models centered on the client, rather than the pathology (D21, D32, D28). Compliance of the patient with the rating system (D11) or difficulty in understanding the scales (D9), and the rating system being difficult for the occupational therapists to explain, was also limitations for these practitioners. Patients' limited abilities were highlighted by this group of experts, particularly around issues of carrying out the COPM due to difficulties of cognition and denial on the part of the patient (D14, D15, D12).

Group E2 (4/33): this group of occupational therapists highlights both the strengths of the scale as a tool to reevaluate the benefits of the rehabilitation program (A23) and also the COPM's framework guiding the interview (A27). For this group, the COPM has value in identifying the role of the occupational therapist within a team (A22) as well as providing elements that contribute to team's decision-making for treatment objectives (A31). Lastly, the scale enables the client to autoevaluate (A13), puts forward the perspective of the client (A4), encourages discussion (A6), and puts responsibility firmly with both the patient and the occupational therapist (A17).

Group E3 (12/33): the occupational therapists in this group were disappointed with the lack of current French research on COPM. For them, the doctor's (D19) and the team's (D10, D16) approaches could be viewed as limitations. Lack of knowledge around the work carried out by occupational therapists (D20) and determination that the focus should be on rehabilitation (D23) were combining factors for this group in terms of understanding the COPM. In contrast, they also felt that the scale gives meaning to the work carried out by occupational therapists (A46), facilitates a psychosocial approach (A45), and encourages team-working (A44) and joint goal setting (A27).

From the second round, all 33 occupational therapists agreed on the benefits of the COPM, whilst having different ideas on the limitations. The graph of items shows some heterogeneity in opinion with regards to limitations. Consensus in the second round of the Delphi survey evidences five groups of benefits. The first group of benefits is in relation to the use of the COPM as a tool to showcase an approach centered on occupation, 81.8% (*n* = 27). This element is linked to the benefits of moving away from a system of reasoning based on pathology, with 72.7% (*n* = 24) in complete agreement 18.2% (*n* = 6) in agreement. The use of the COPM encourages a psychosocial approach for 87.9% (*n* = 29). The second group of benefits highlights the COPM as an interview that facilitates the capture of the patient's voice 97.0% (*n* = 32), giving space for the person to express their wishes 87.9% (*n* = 29), encourages discussion 87.9% (*n* = 29), gives the person a leading voice 90.9% (*n* = 30), and also takes into account the person's environment 84.8% (*n* = 28). The third group of benefits was centered around the possible impact on the person by encouraging their engagement and their engagement 97.0% (*n* = 32). Here, we found evidence of the principal objectives of the COPM: identifying the specific difficulties of the person 97.0% (*n* = 32) and enabling to understand the daily difficulties of the person interviewed 93.9% (*n* = 31). For 90.9% (*n* = 30), the COPM facilitates habilitation for the person interviewed and the therapist, as well as negotiation between them 93.9% (*n* = 31). The fourth group of benefits is centered around the operational side of the measure; notably evaluating performance and satisfaction is important for 97.0% (*n* = 32) of the occupational therapists participating in this study, and the COPM enables them to put in place negotiated goals and gives the client the power to decide on their goals 93.9% (*n* = 31). This tool was highlighted as being effective in measuring outcomes of the intervention 93.9% (*n* = 31) and for reevaluation 90.9% (*n* = 30). For 97.0% (*n* = 32) of the participants, the measure facilitates the formulation of an occupational therapy diagnosis, provides a strong argument for team decision making 90.9% (*n* = 30), and supports team working 84.8% (*n* = 28). The final group of benefits is linked to habilitation, in that the COPM promotes habilitation for the person and the therapist, to varying degrees of “completely agree” 51.5% (*n* = 17) and “agree” 39.4% (*n* = 13). Habilitation is also associated with several other elements for the participants, specifically concerned with the fostering or encouragement of negotiation between the client and the occupational therapist 93.9% (*n* = 31), taking a decision 97.0% (*n* = 32) and decision-making for clients 90.9% (*n* = 30). For all experts 100% (*n* = 33), the use of the COPM can provide meaning to the occupational therapists' practice in their work with 60.6% (*n* = 20) in complete agreement and 33.3% (*n* = 11) in agreement. Finally, for 87.9% (*n* = 29), the use of the COPM supports a psychosocial approach.

In terms of limitations, lack of insight and denial figured highly 57.6% (*n* = 19) (“completely agree”) and slightly less 24.2% (*n* = 8) (“agree”). Problems in relation to attention, understanding, and significant behavioral difficulties are obstacles in the use of the COPM 84.8% (*n* = 28), accompanied by a perception that communication difficulties can present a barrier 87.9% (*n* = 29). French institutional systems are highlighted as a barrier to the use of this tool 81.8% (*n* = 27), with a lack of knowledge of the model gained from preregistration training also being underlined as a difficulty, 45.5% (*n* = 15) completely agreed and 21.2% (*n* = 7) agreed.

### 3.4. Third Consultation with the Experts

Following the calculation of percentages, 32 benefits and 5 limits were carried into the final phase. To form the final list, only statements with 80% or more agreement are addressed in the discussion. The result gives 32 interests and 1 limit to the use of the CORM for experts (Table 3).

The majority of the discriminating items (20/28), which enabled the PCA-based definition of the three expert groups during the second round, were set aside due to a consensus below 80%. Thereby, the influence of these group-specific lines of thinking was eliminated in the PCA analysis of the third-round results, that represent accordingly the influence of less discriminative items. But although being less distinctive (highest vector length < 0.5 (Figure 5(b)) compared to >1 in the 2^nd^ round (Figure 4(b))), they could still influence the consensus achieved in this final round. The analysis of the item projections confirms that consensus was reached for the benefits of the tool (Figure 5(b)). In fact, the majority of the corresponding items (25/31 benefits) have weights (vector lengths) that are very weak or null (between 0.00 and 0.07), and only 3 of them have weak weights, from 0.09 to 0.20, signifying no and little variability, respectively. With respect to the limitations, there was consensus on only one item (1/5 limitations), with a weak weight of 0.10. Only 7 items (4 limitations and 3 benefits) showed a stronger divergence, their weights being between 0.21 and 0.39. These were the key factors in the definition of groups for the third round (Figure 5(b)). In fact, on the PCA graph (Figure 5(a)), three groups are clearly distinguishable due to these discriminating items (A14, A18, A20, D3, D13, D15, D32). While group EI (25 individuals) agreed on all of them, they were completely rejected by group EIII (2 individuals). Group EII (6 individuals) took intermediate position, agreeing on the items A14, A18, and A20, but also distinguishing itself by being in disagreement on items D3, D13, D15, and D32. The final outcome of this Delphi study demonstrates a consensus of 31 benefits and 1 limitation for the use of the COPM, according to French occupational therapy experts.

## 4. Discussion and Implications

The objective of this study was to establish a baseline of current use in practice of the COPM by consulting 33 expert French occupational therapists. The PCA analysis revealed the occupational therapists' predominant themes in thinking based on their responses in the second and third rounds of the Delphi study. The results are discussed below in the context of the respective international literature.

The PCA analysis from the second round permitted the classification of the experts, corresponding to three different profiles in groups E1, E2, E3:

Group E1 (17/33) mirrored the institutional and conceptual limitations that can be found in the French health sector. Indeed, the CMOP-E appears to be an indispensable model for occupational therapy practice in Anglo-Saxon countries [13] but struggles to find its place in French-speaking countries (with the exception of Quebec from whence the COPM was developed).

The stance of group E2 (4/33) revealed a commitment to using the COPM, highlighting the importance of the principles of this method that are widely cited in the literature [6]. Accordingly, when assessing patient performance, occupational therapists must take into account their individual and unique needs and abilities. Also, group E2 attached much importance to the opportunity to rate performance and satisfaction as a mean to orientate the therapeutic intervention, which is in accordance with COPM being a valid, trustworthy, adaptable, and clinically useful outcome measure, both for patients and their caregivers [14]. Further, for group E2, the COPM promotes the clients' engagement in the process of rehabilitation with a sense of shared responsibility. A recent single case study [14] demonstrated the benefits of the COPM for people who have multiple sclerosis and their engagement. For this group, the COPM was presented as an interview that permits the recording of the client's point of view, to foster dialogue, to give space to the expression of desires, and to consider the client's environment. This benefit of the COPM has also been demonstrated in studies with people who have had a CVA [15], chronic pain [16] or those with spinal cord injury [17].

Group E3 (12/33) highlighted the lack of compelling evidence in the French language and the paucity of studies in France on this subject. Groups E3 and E1 are linked in their perceived difficulties in taking a client-centered occupational approach in the face of issues related to organization, institutions, and acceptance on the part of interprofessional health teams to adopt a change of their practice model. Progression in Canadian health and social care policies proves the importance of solid evidence, results and analysis, and models of occupational therapy practice that promote well-being, habilitation, and justice founded on occupation [18]. By clearly demonstrating a positive contribution of COPM in the occupational therapy, this Delphi study should strengthen the acceptance of this method in the French healthcare system.

In addition, the PCA analysis from the third Delphi round brought out three profiles of participants experts EI, EII, and EIII that are described in the following:

EI, the largest group, composed of 25/33 individuals, agreed with all items retained from the second round, i.e., 31 benefits as well as 5 limitations, and presents thereby the most complete consensus profile on the use of the COPM in France. Most discriminating with respect to the other groups was that EI did not reject any limitation. Thus, for this large part of experts, institutional systems, and lack of knowledge of client-centered, occupational models can be barriers to the use of the COPM in France.

Group EII, composed of 6 individuals, is distinguished from group EI by a disagreement on four out of five limitations. Institutional systems and lack of knowledge about the model are not held to be limitations for this group. These limitations may impact less when an occupational therapist works in private or independent practice, or in an institution that is already structured around the values of the CMOP-E model. Further, for clients who have a lack of insight or denial, Phipps and Richardson's study [19] supports group EII's positive perspective, demonstrating a significant improvement in this population in relation to awareness of impairments after outpatient treatment using the COPM, and thereby questioning the perception of barriers to the use of the COPM with this population.

Group EIII (*n* = 2) is distinguished from the other groups by rejecting the four limitations from group EII and in rejecting the idea that the COPM creates an environment where giving a voice to the person and enabling expression of desires is possible. Law et al. [2] showed that since 1991, the COPM was primarily seen as a method, permitting identification of problem areas in performance and satisfaction and to measure changes perceived by the person following occupational therapy and interprofessional interventions. According to these authors, expression of desires is not a priority of the COPM, and giving the patients a voice is focused on everyday difficulties.

The only limit that has been retained is disorders related to attention, comprehension, or behavior (D14). In fact, a literature review shows that the COPM is not well adapted for clients who have difficulties in planning ahead, in decision-making for themselves or others [20]. Studies, mainly in the mental health sector, suggest using the COPM with caution since this method may be discouraging for the client if he/she cannot perceive the subtleties of rating achievements and satisfaction [21]. For all experts (100%), the benefit of using the COPM is its person-centered approach, reinforcing the role and participation of the client (A17, A25, A29, A30, A37, A38) and breaking with pathology-based approaches (A39). Using the COPM facilitates an objective rating of progression in performance and satisfaction (A19, A24, A40). Finally, the COPM creates an environment that renders possible the negotiation between therapist and client (A3). Even in 1993, Pollock [22] emphasized the benefit of using COPM to centre therapeutic practice on issues related to the occupations of the client. With very high consensus, although not unanimous, these experts validated the following items: 97% (*n* = 32) of the occupational therapists were in agreement with the occupational aspects of the tool (A1). In fact, increasing research in occupational science puts forward tangible evidence of the effectiveness of occupational therapy practice [23]. In all, 97% (*n* = 32) of the experts agreed that the COPM brings meaning into the practice of occupational therapy (A46). Morrison et al. [24] reviewed the development of occupational science based on scientific publications from 2001 to 2012. The authors identified two axes for the use of occupational science. In the first axis, occupation is considered a practical way to describe humankind, the behavior of people in society, and the possibility for changes when facing disability. Additionally, the first axis takes into account also the development of professional practice in occupational therapy. In the second axis, occupational science is seen as study subject in order to consolidate the conceptual approaches in this domain. The COPM provides the opportunities to make choices and fosters the client's decision-making, further providing the opportunity to negotiate and jointly conceive a treatment plan. Indeed, since the year 2000, studies have demonstrated that, with the COPM, clients feel empowered to name their difficulties and improve their life habits and the circumstances that lead to those difficulties [25]. For the last 7 items, consensus was between 91% and 88%. In particular, the COPM was identified as effective in reevaluating the elements of the client's performance and satisfaction (A2, A3, A21), as well as in promoting habilitation (A20). This concept, first described by the World Health Organization in 1986 as an approach that fosters conferring the decision-making power to the individual, has been taken by Townsend and Polatajko [4] to illustrate occupational justice. Drolet [26] demonstrated that habilitation is supported by establishing an environment that facilitates ability, as well as the capacity of being and doing.

The anonymity demanded by the Delphi technique protocols did not allow us to identify the experts between each stage, and hence it was not possible to deepen the PCA analysis to study, for example, the progression of opinions of each occupational therapist through the three Delphi rounds. An adapted form of the Delphi method could be envisaged, in order to study not only group consensus but also the individual's positions taken in relation to the questions posed and their progression through each stage.

Finally, with respect to interprofessional collaborations, it would be interesting to assess whether outcome measures used by other professionals could be complementary to the COPM or whether the joint use of common tools could be relevant. In France, the COPM is an outcome measure requiring CPD training, available only to occupational therapists. What about other tools from other professionals? If the COPM is pertinent for the development of interprofessional treatment planning, then could this method not be the single assessment for the interprofessional team? And the occupational therapist therefore be a coordinator of occupational issues on behalf of the client?

## 5. Conclusion

The results of this Delphi study and the analysis of the principal components demonstrate a consensus of occupational therapists on the benefits and limitations of the use of the COPM in France. The consensus of these experts shows that the use of the COPM within the French health system has many advantages and few disadvantages. Therefore, there appears to be neither a significant obstacle to expanding the use of this method nor significant differences in the use of this measure in Canada or Anglo-Saxon countries. This outcome measure fosters an approach centered on the person and their caregivers by determining the range of problematic occupations and the objectives which provide the key elements of the treatment plan. The COPM gives occupational therapist the opportunity to use a reliable tool to establish objectives and rehabilitation plans in the interprofessional teams. This interview provides space for the client, enabling them to actively collaborate and participate in their rehabilitation process. However, it seems crucial to invest more into research and into the dissemination of information about the strong evidence for the use of the COPM, in order to increase health sector knowledge of the tool, that of the occupational therapy client-centered approach and of occupational science. An emphasis on the training of occupational therapists in the use of the tool is imperative both in preregistration and CPD training. The validation of the specific role of occupational therapy in relation to coordination and rehabilitation needs to be reinforced, as well as increased knowledge on the concept of habilitation as elucidated in the Canadian Model of Occupational Performance and Engagement (CMOP-E). This model and its outcome measure focus interventions on occupation, a central tenet for occupational therapists internationally and increasingly in French speaking countries. This research should be followed by complementary studies focused on the use of the COPM in French-speaking countries, the study of its effects on the practice of the occupational therapist, and the development of occupations for the client.

## Figures and Tables

**Table 1:** Items retained following the first expert consultation.

Interests	Limits
Favors the occupancy-centered approach Identifies the person's specific problems Promotes dialogue Enables confidence building Evaluates performance and satisfaction Studies the activities in the usual living context Promotes the negotiated implementation of objectives Gives the person a voice gives the caregiver a voice Promotes the therapist's positioning Promotes the person's decision Allows the person to understand the problems of the person's daily life Puts the person back at the centre of concerns of caregivers Provides a reassessment Promotes awareness of everyday problems Considers the environment through the person's discourse Identifies the person's representations Provides arguments for team syntheses Gives a rating of the importance of the difficulty of performance and satisfaction Encourages the person's commitment Encourages collaborative work Allows for self-evaluation and self-assessment. Evaluation for users Allows for the framing of the practice Allows for the measurement of the results of the intervention Encourages the person's involvement Gives the person back the power to decide on his or her objectives Allows for the targeting of objectives for the entire team Participates in the occupational therapy diagnosis Allows for the needs assessment of people and activities that make sense Allows for the implementation of negotiated objectives Gives a framework to guide the interview Objectively evaluates the impact of the intervention Helps to identify the role/area of the occupational therapist Promotes the implementation of COOP Leaves a space for exchange and expression of one's desires Enables empowerment of the person and the occupational therapist Allows the client's voice to be heard (person, group, organization) Opens up new areas of support	Pathocentric approach Organizational system of the institutes Institutional functioning Ignorance of our actions Ignorance of the occupation-centered approach The financing and reimbursement system Presence of certain biases depending on the therapist's attitude Difficulty in elaborating or making quotations for patients Poor understanding of the scales by the patient The support of the whole team Patient adherence to the numerical scoring system Patients wishing to recover lost functions Communication disorders Attention, comprehension, or major behavioral problems Anosognosia or denial of difficulties Refusal or passive opposition of the team to this tool Failure to return the patient home prior to assessment Lifestyle habits that indirectly affect his health. The goals most often set by the doctor or therapist Lack of knowledge by occupational therapists in France Ignorance of actions centered on the needs of the person The time spent for the interview The certainty in France that rehabilitation must come first The great difficulty of accepting disability Lack of supervision and monitoring The discrepancy with the request of the family and friends and the person concerned Adapted question formulation The use of the word “occupation” The quotation is difficult to explain The apathy of people to integrate this approach What little French scientific data there is on the subject Ignorance of these models in initial training

**Table:** (a) Results of the second round of consultation on the COPM benefits

Statements/interests	Average score	*T*-difference
A1 favors the occupational-centered approach	4.00	0
A28 participates in the occupational therapy diagnosis	3.70	0.68
A29 allows to start from the needs of activities that make sense	3.64	0.78
A2 identifies the person's specific problems	3.61	0.79
A7 allows for the implementation of negotiated objectives	3.61	0.79
A26 provides arguments for team syntheses	3.57	0.56
A19 evaluates performance and satisfaction	3.55	0.79
A35 opens up the accompaniment to new areas	3.55	0.79
A5 provides a rating of performance and satisfaction	3.52	0.80
A25 encourages the involvement of the person	3.52	0.80
A38 restores the power to decide on its objectives	3.52	0.83
A39 allows to get out of a logic centered on the pathology	3.52	0.87
A40 - allows the results of the intervention to be measured	3.48	0.76
A24 allows the client's voice to be heard	3.48	0.80
A37 promotes the person's commitment	3.45	0.79
A8 gives the opportunity to make choices	3.45	0.79
A18 gives the floor to the person	3.42	0.87
A46 gives meaning to the occupational therapist's work	3.42	0.83
A3 promotes negotiation between client/occupational therapist	3.39	0.75
A36 allows therapeutic orientations	3.34	0.75
A20 enables the habilitation	3.36	0.78
A30 promotes the person's decision	3.34	0.78
A12 allows to understand the problems of everyday life	3.33	0.74
A44 facilitates teamwork	3.33	0.92
A17 empowers the client and the occupational therapist	3.33	0.78
A21 ensures a reassessment	3.30	0.92
A14 leaves a space for the expression of one's desires	3.27	0.88
A6 promotes dialogue	3.24	0.79
A11 studies the activities in the context of the usual life	3.24	0.83
A45 favors the psychosocial approach	3.24	0.79
A43 considers the environment through discourse	3.21	0.78
A4 identifies the representations of the person	3.21	0.82
A10 encourages collaborative work	3.18	0.81
A16 takes into account the person's environment	3.15	0.71
A41 puts the person back at the center of attention	3.12	0.86
A42 promotes awareness of problems	3.12	0.78
A15 promotes the positioning of the therapist	3.09	0.84
A32 allows for confidence building	3.06	0.90
A23 objectively evaluates the impact of the intervention	3.06	0.86
A27 allows for the framing of the practice	3.00	1.00
A31 targets objectives for the whole team	3.00	0.79
A33 provides a framework to guide the interview	3.00	0.75
A22 helps to identify the role/domain of the occupational therapist	3.00	0.95
A13 allows self-assessment for users	2.94	0.97
A9 gives voice to caregivers	2.64	0.82
A34 encourages the establishment of COOP	2.36	0.93

**Table:** (b) Results of the second round of consultation on the COPM limitations

Statements/limits	Average score	*T*-difference
D14-attention, comprehension, or major behavioral disorders	3.27	0.91
D15-anosognosia or denial of difficulties	3.24	1.00
D3-institutional functioning	3.20	0.58
D13-communication disorders	3.20	0.77
D32-lack of knowledge of these models in initial training	3.20	0.92
D19-the goals are most often set by the doctor or therapist	2.94	1.03
D23-the certainty in France that reeducation must come first	2.94	0.95
D5-lack of awareness of the occupation-centered approach	2.91	1.10
D8-difficulty in developing or making quotations for some patients	2.91	1.10
D2-institute organizational system	2.82	0.95
D31-the lack of French-language scientific data on the subject	2.79	1.05
D30-the apathy of the people to integrate this approach	2.73	0.88
D20-lack of knowledge by occupational therapists in France	2.64	1.03
D12-patients wishing to recover lost functions	2.61	1.17
D9-patient misunderstanding of scales	2.58	1.15
D21-ignorance of actions centered on the needs of the individual	2.58	0.97
D25-lack of supervision and monitoring	2.58	0.83
D7-presence of certain biases depending on the therapist's attitude	2.52	1.09
D10-the adhesion of the whole team	2.48	0.97
D1-pathocentric approach	2.45	1.18
D4-ignorance of occupational therapist's actions	2.45	1.09
D26-the discrepancy with the request of the entourage and the person concerned	2.42	1.06
D27-appropriate question wording	2.42	1.00
D28-the use of the word “occupation”	2.39	1.12
D11-patient adherence to the numerical scoring system	2.36	0.99
D24-the difficulty of accepting disability	2.27	1.04
D6-the financing and reimbursement system	2.24	0.97
D29-the rating is difficult to explain	2.24	1.15
D16-passive opposition of the team to this tool	2.18	0.95
D22-time spent on maintenance	2.12	0.89
D17-failure to return the patient home prior to assessment	2.09	1.07
D18-lifestyle habits that indirectly harm his health	1.94	0.93

**Table 3:** Results of the third round of consultation on the COPM benefits and limitations.

Statements/interests and limits	*n*	%
Interests	33	100
A3 promotes negotiation between client/occupational therapist A8 gives the opportunity to make choices A17 empowers the client and the occupational therapist A19 evaluates performance and satisfaction A24 allows the client's voice to be heard A25 encourages the involvement of the person A29 allows to start from the needs of activities that make sense A30 promotes the person's decision A37 promotes the person's commitment A38 restores the power to decide on its objectives A39 allows to get out of a logic centered on the pathology A40 allows the results of the intervention to be measured		
A1 favors the occupational-centered approach A4 identifies the representations of the person A28 participates in the occupational therapy diagnosis A46 gives meaning to the occupational therapist's work A6 promotes dialogue	32	97
A7 allows for the implementation of negotiated objectives A12 allows to understand the problems of everyday life A26 provides arguments for team syntheses A35 opens up the accompaniment to new areas A36 allows therapeutic orientations A44 facilitates teamwork A45 favors the psychosocial approach	31	94
A2 identifies the person's specific problems A5 provides a rating of performance and satisfaction	30	91
A11 studies the activities in the context of the usual life A14 leaves a space for the expression of one's desires A18 gives the floor to the person A20 enables the habilitation A21 ensures a reassessment	29	88
	29	88
Limits		
D14-attention, comprehension, or major behavioral disorders	32	97

## Data Availability

The data used to support the findings of this study are available from the first author (Jean-Michel Caire) upon request.

## Supplementary Materials

Supplementary Materials SD1: demographic elements of the expert population. SD2: items retained following the first expert consultation. SD3: results of the second round of consultation on the COPM benefits. SD4 : results of the second round of consultation on the COPM limitations. SD5: results of the third round of consultation on the COPM benefits and limitations. SD6: results of the third round of consultation on the COPM benefits and limitations.
