# Supplementary material for: Canadian Occupational Performance Measure: Benefits and Limitations Highlighted Using the Delphi Method and Principal Component Analysis
Source: Occup Ther Int. 2022 Mar 2;2022:9963030. doi: 10.1155/2022/9963030 (PMC8906980; doi:10.1155/2022/9963030)
Supplement: Supplementary Materials — SD1: demographic elements of the expert population. SD2: items retained following the first expert consultation. SD3: results of the second round of consultation on the COPM benefits. SD4 : results of the second round of consultation on the COPM limitations. SD5: results of the third round of consultation on the COPM benefits and limitations. SD6: results of the third round of consultation on the COPM benefits and limitations. [file 9963030.f1.docx]

**Supplementary materials**

SD1 - Demographic elements of the expert population

SD2 - Items retained following the first expert consultation

| INTERESTS | LIMITS |
| --- | --- |
| Favours the occupancy-centred approach  Identifies the person's specific problems  Promotes dialogue  Enables confidence building  Evaluates performance and satisfaction  Studies the activities in the usual living context  Promotes the negotiated implementation of objectives  Gives the person a voice Gives the caregiver a voice  Promotes the therapist's positioning  Promotes the person's decision  Allows the person to understand the problems of the person's daily life  Puts the person back at the centre of concerns of caregivers  Provides a reassessment  Promotes awareness of everyday problems  Considers the environment through the person's discourse  Identifies the person's representations  Provides arguments for team syntheses  Gives a rating of the importance of the difficulty of performance and satisfaction.  Encourages the person's commitment  Encourages collaborative work  Allows for self-evaluation and self-assessment.  Evaluation for users  Allows for the framing of the practice  Allows for the measurement of the results of the intervention  Encourages the person's involvement  Gives the person back the power to decide on his or her objectives  Allows for the targeting of objectives for the entire team  Participates in the occupational therapy diagnosis  Allows for the needs assessment of people and activities that make sense  Allows for the implementation of negotiated objectives  Gives a framework to guide the interview  Objectively evaluates the impact of the intervention  Helps to identify the role/area of the occupational therapist  Promotes the implementation of COOP  Leaves a space for exchange and expression of one's desires,  Enables empowerment of the person and the occupational therapist  Allows the client's voice to be heard (person, group, organisation)  Opens up new areas of support, | Patho-centric approach  Organizational System of the Institutes  Institutional functioning  Ignorance of our actions  Ignorance of the occupation-centred approach  The financing and reimbursement system  Presence of certain biases depending on the therapist's attitude.  Difficulty in elaborating or making quotations for patients.  Poor understanding of the scales by the patient  The support of the whole team  Patient adherence to the numerical scoring system  Patients wishing to recover lost functions  Communication disorders  Attention, comprehension or major behavioural problems Anosognosia or denial of difficulties  Refusal or passive opposition of the team to this tool  Failure to return the patient home prior to assessment  Lifestyle habits that indirectly affect his health.  The goals most often set by the doctor or therapist  Lack of knowledge by occupational therapists in France  Ignorance of actions centred on the needs of the person  The time spent for the interview  The certainty in France that rehabilitation must come first  The great difficulty of accepting disability  Lack of supervision and monitoring  The discrepancy with the request of the family and friends and the person concerned.  Adapted question formulation  The use of the word "occupation"  The quotation is difficult to explain  The apathy of people to integrate this approach  What little French scientific data there is on the subject  Ignorance of these models in initial training |

SD3 - Results of the second round of consultation on the COPM benefits

| **Statements / Interests** | **Average score** | **T-difference** |
| --- | --- | --- |
| A1 - Favours the occupational-centred approach | 4,00 | 0 |
| A28 - Participates in the occupational therapy diagnosis | 3,70 | 0,68 |
| A29 - Allows to start from the needs of activities that make sense | 3,64 | 0,78 |
| A2 - Identifies the person's specific problems | 3,61 | 0,79 |
| A7 - Allows for the implementation of negotiated objectives | 3,61 | 0,79 |
| A26 - Provides arguments for team syntheses | 3,57 | 0,56 |
| A19 - Evaluates performance and satisfaction | 3,55 | 0,79 |
| A35 - Opens up the accompaniment to new areas | 3,55 | 0,79 |
| A5 - Provides a rating of performance and satisfaction | 3,52 | 0,80 |
| A25 - Encourages the involvement of the person | 3,52 | 0,80 |
| A38 - Restores the power to decide on its objectives | 3,52 | 0,83 |
| A39 - Allows to get out of a logic centered on the pathology | 3,52 | 0,87 |
| A40 - Allows the results of the intervention to be measured | 3,48 | 0,76 |
| A24 - Allows the client's voice to be heard | 3,48 | 0,80 |
| A37 - Promotes the person's commitment | 3,45 | 0,79 |
| A8 - Gives the opportunity to make choices | 3,45 | 0,79 |
| A18 - Gives the floor to the person | 3,42 | 0,87 |
| A46 - Gives meaning to the occupational therapist's work | 3,42 | 0,83 |
| A3 - Promotes negotiation between client/occupational therapist | 3,39 | 0,75 |
| A36 - Allows therapeutic orientations | 3,34 | 0,75 |
| A20 - Enables the habilitation | 3,36 | 0,78 |
| A30 - Promotes the person's decision | 3,34 | 0,78 |
| A12 - Allows to understand the problems of everyday life | 3,33 | 0,74 |
| A44 - facilitates teamwork | 3,33 | 0,92 |
| A17 - Empowers the client and the occupational therapist | 3,33 | 0,78 |
| A21 - Ensures a reassessment | 3,30 | 0,92 |
| A14 - Leaves a space for the expression of one's desires | 3,27 | 0,88 |
| A6 - Promotes dialogue | 3,24 | 0,79 |
| A11 - Studies the activities in the context of the usual life | 3,24 | 0,83 |
| A45 - Favours the psycho-social approach | 3,24 | 0,79 |
| A43 - Considers the environment through discourse | 3,21 | 0,78 |
| A4 - Identifies the representations of the person | 3,21 | 0,82 |
| A10 - Encourages collaborative work | 3,18 | 0,81 |
| A16 - Takes into account the person's environment | 3,15 | 0,71 |
| A41 - Puts the person back at the centre of attention | 3,12 | 0,86 |
| A42 - Promotes awareness of problems | 3,12 | 0,78 |
| A15 - Promotes the positioning of the therapist | 3,09 | 0,84 |
| A32 - Allows for confidence building | 3,06 | 0,90 |
| A23 - Objectively evaluates the impact of the intervention | 3,06 | 0,86 |
| A27 - Allows for the framing of the practice | 3,00 | 1,00 |
| A31 - Targets objectives for the whole team | 3,00 | 0,79 |
| A33 - Provides a framework to guide the interview | 3,00 | 0,75 |
| A22 - Helps to identify the role/domain of the occupational therapist | 3,00 | 0,95 |
| A13 - Allows self-assessment for users | 2,94 | 0,97 |
| A9 - Gives Voice to Caregivers | 2,64 | 0,82 |
| A34 - Encourages the establishment of COOP | 2,36 | 0,93 |

SD4 - Results of the second round of consultation on the COPM limitations

| **Statements / Limits** | **Average score** | **T-difference** |
| --- | --- | --- |
| D14 - Attention, comprehension or major behavioural disorders | 3,27 | 0,91 |
| D15 - Anosognosia or denial of difficulties | 3,24 | 1,00 |
| D 3 - Institutional functioning | 3,20 | 0,58 |
| D 13 - Communication disorders | 3,20 | 0,77 |
| D 32 - Lack of knowledge of these models in initial training | 3,20 | 0,92 |
| D 19 - The goals are most often set by the doctor or therapist | 2,94 | 1,03 |
| D 23 - The certainty in France that re-education must come first | 2,94 | 0,95 |
| D 5 - Lack of awareness of the occupation-centred approach | 2,91 | 1,10 |
| D 8 - Difficulty in developing or making quotations for some patients | 2,91 | 1,10 |
| D 2 - Institute organizational system | 2,82 | 0,95 |
| D 31 - The lack of French-language scientific data on the subject | 2,79 | 1,05 |
| D 30 - The apathy of the people to integrate this approach | 2,73 | 0,88 |
| D 20 - Lack of knowledge by occupational therapists in France | 2,64 | 1.03 |
| D 12 - Patients wishing to recover lost functions | 2,61 | 1,17 |
| D 9 - Patient misunderstanding of scales | 2,58 | 1,15 |
| D 21 - Ignorance of actions centred on the needs of the individual | 2,58 | 0,97 |
| D 25 - Lack of supervision and monitoring | 2,58 | 0,83 |
| D 7 - Presence of certain biases depending on the therapist's attitude | 2,52 | 1,09 |
| D 10 - The adhesion of the whole team | 2,48 | 0,97 |
| D 1 - Patho-centric approach | 2 ,45 | 1,18 |
| D 4 - Ignorance of occupational therapist’s actions | 2,45 | 1,09 |
| D 26 - The discrepancy with the request of the entourage and the person concerned | 2,42 | 1,06 |
| D 27 - Appropriate question wording | 2,42 | 1,00 |
| D 28 - The use of the word "occupation" | 2,39 | 1,12 |
| D 11 - Patient adherence to the numerical scoring system | 2,36 | 0,99 |
| D 24 - The difficulty of accepting disability | 2,27 | 1,04 |
| D 6 - The financing and reimbursement system | 2,24 | 0,97 |
| D 29 - The rating is difficult to explain | 2,24 | 1,15 |
| D 16 - Passive opposition of the team to this tool | 2,18 | 0,95 |
| D 22 - Time spent on maintenance | 2,12 | 0,89 |
| D 17 - Failure to return the patient home prior to assessment | 2,09 | 1,07 |
| D 18 - Lifestyle habits that indirectly harm his health. | 1,94 | 0,93 |

SD5 - Results of the third round of consultation on the COPM benefits and limitations

| **Statements / Interests and limits** | **n** | **%** |
| --- | --- | --- |
| **Interests**  A3 - Promotes negotiation between client/occupational therapist  A8 - Gives the opportunity to make choices  A17 - Empowers the client and the occupational therapist  A19 - Evaluates performance and satisfaction  A24 - Allows the client's voice to be heard  A25 - Encourages the involvement of the person  A29 - Allows to start from the needs of activities that make sense  A30 - Promotes the person's decision  A37 - Promotes the person's commitment  A38 - Restores the power to decide on its objectives  A39 - Allows to get out of a logic centered on the pathology  A40 - Allows the results of the intervention to be measured  A1 - Favours the occupational-centred approach  A4 - Identifies the representations of the person  A28 - Participates in the occupational therapy diagnosis  A46 - Gives meaning to the occupational therapist's work  A6 – Promotes dialogue  A7 - Allows for the implementation of negotiated objectives  A12 - Allows to understand the problems of everyday life  A26 - Provides arguments for team syntheses  A35 - Opens up the accompaniment to new areas  A36 - Allows therapeutic orientations  A44 - facilitates teamwork  A45 - Favours the psycho-social approach  A2 - Identifies the person's specific problems  A5 - Provides a rating of performance and satisfaction  A11 - Studies the activities in the context of the usual life  A14 - Leaves a space for the expression of one's desires  A18 - Gives the floor to the person  A20 - Enables the habilitation  A21 - Ensures a reassessment  **Limits**  D14 - Attention, comprehension or major behavioural disorders | 33  32  31  30  29  29  32 | 100  97  94  91  88  88  97 |

SD6 - Results of the third round of consultation on the COPM benefits and limitations

| **Statements / Interests and limits** | **n** | **%** |
| --- | --- | --- |
| **Interests**  A3 - Promotes negotiation between client/occupational therapist  A8 - Gives the opportunity to make choices  A17 - Empowers the client and the occupational therapist  A19 - Evaluates performance and satisfaction  A24 - Allows the client's voice to be heard  A25 - Encourages the involvement of the person  A29 - Allows to start from the needs of activities that make sense  A30 - Promotes the person's decision  A37 - Promotes the person's commitment  A38 - Restores the power to decide on its objectives  A39 - Allows to get out of a logic centered on the pathology  A40 - Allows the results of the intervention to be measured  A1 - Favours the occupational-centred approach  A4 - Identifies the representations of the person  A28 - Participates in the occupational therapy diagnosis  A46 - Gives meaning to the occupational therapist's work  A6 – Promotes dialogue  A7 - Allows for the implementation of negotiated objectives  A12 - Allows to understand the problems of everyday life  A26 - Provides arguments for team syntheses  A35 - Opens up the accompaniment to new areas  A36 - Allows therapeutic orientations  A44 - facilitates teamwork  A45 - Favours the psycho-social approach  A2 - Identifies the person's specific problems  A5 - Provides a rating of performance and satisfaction  A11 - Studies the activities in the context of the usual life  A14 - Leaves a space for the expression of one's desires  A18 - Gives the floor to the person  A20 - Enables the habilitation  A21 - Ensures a reassessment  **Limits**  D14 - Attention, comprehension or major behavioural disorders | 33  32  31  30  29  29  32 | 100  97  94  91  88  88  97 |
